# Tacrolimus-Loaded Solid Lipid Nanoparticle Gel: Formulation Development and In Vitro Assessment for Topical Applications

**DOI:** 10.3390/gels8020129

**Published:** 2022-02-18

**Authors:** Abdul Shakur Khan, Kifayat Ullah Shah, Mohammed Al Mohaini, Abdulkhaliq J. Alsalman, Maitham A. Al Hawaj, Yousef N. Alhashem, Shakira Ghazanfar, Kamran Ahmad Khan, Zahid Rasul Niazi, Arshad Farid

**Affiliations:** 1 Faculty of Pharmacy, Gomal University, Dera Ismail Khan 29050, Pakistan; abdulshakurkhan01@gmail.com (A.S.K.); dr.kamrangu@gmail.com (K.A.K.); zahidscholar1@gmail.com (Z.R.N.); 2 Basic Sciences Department, College of Applied Medical Sciences, King Saud bin Abdulaziz University for Health Sciences, Alahsa 31982, Saudi Arabia; mohainim@ksau-hs.edu.sa; 3 King Abdullah International Medical Research Center, Alahsa 31982, Saudi Arabia; 4 Department of Clinical Pharmacy, Faculty of Pharmacy, Northern Border University, Rafha 91911, Saudi Arabia; kaliqs@gmail.com; 5 Department of Pharmacy Practice, College of Clinical Pharmacy, King Faisal University, Ahsa 31982, Saudi Arabia; hawaj@kfu.edu.sa; 6 Clinical Laboratory Sciences Department, Mohammed Al-Mana College for Medical Sciences, Dammam 34222, Saudi Arabia; yousefa@machs.edu.sa; 7 National Institute for Genomics Advanced Biotechnology, National Agricultural Research Centre, Park Road, Islamabad 45500, Pakistan; shakira_akmal@yahoo.com; 8 Gomal Center of Biochemistry and Biotechnology, Gomal University, Dera Ismail Khan 29050, Pakistan

**Keywords:** tacrolimus, chitosan, solid lipid nanoparticles, gel, topical drug delivery

## Abstract

The currently available topical formulations of tacrolimus have minimal and variable absorption, elevated mean disposition half-life, and skin irritation effects resulting in patient noncompliance. In our study, we fabricated tacrolimus-loaded solid lipid nanoparticles (SLNs) that were converted into a gel for improved topical applications. The SLNs were prepared using a solvent evaporation method and characterized for their physicochemical properties. The particle size of the SLNs was in the range of 439 nm to 669 nm with a PDI of ≤0.4, indicating a monodispersed system. The Zeta potential of uncoated SLNs (F1–F5) ranged from −25.80 to −15.40 mV. Those values reverted to positive values for chitosan-decorated formulation (F6). The drug content and entrapment efficiency ranged between 0.86 ± 0.03 and 0.91 ± 0.03 mg/mL and 68.95 ± 0.03 and 83.68 ± 0.04%, respectively. The pH values of 5.45 to 5.53 depict their compatibility for skin application. The surface tension of the SLNs decreased with increasing surfactant concentration that could increase the adherence of the SLNs to the skin. The release of drug from gel formulations was significantly retarded in comparison to their corresponding SLN counterparts (*p* ≤ 0.05). Both SLNs and their corresponding gel achieved the same level of drug permeation, but the retention of the drug was significantly improved with the conversion of SLNs into their corresponding gel formulation (*p* ≤ 0.05) due to its higher bioadhesive properties.

## 1. Introduction

Human skin provides the most comfortably accessible route of drug administration. Topical administration is the route of choice for cutaneous pathologies like atopic dermatitis since it rarely presents systemic adverse effects when compared to other routes of drug administration [1]. The stratum corneum of the skin, composed of flat dead cells encompassing high keratin filaments surrounded by a lipophilic matrix consisting of keratin, ceramides, cholesterol, cholesterol esters, and various other fatty acids, provides a natural physical barrier against particle penetration [2,3]. Many transdermal methods have been tried to overcome the barrier function of the stratum corneum and achieve required transdermal permeability, but nanotechnology has developed an attractive niche in transdermal drug delivery. The physicochemical properties of nanoparticles such as size, shape, viscosity, and surface tension have a significant effect on dermal drug delivery [4], but the importance of nanoparticle composition cannot be underestimated [5].

The SLNs composed of physiological lipids that are solid at room temperature have a generally recognized as safe (GRAS) status for topical application. These carriers combine the advantages of emulsions, liposomes, and polymeric nanoparticles [6]. The SLNs can be used on inflamed skin due to the nonirritating and nontoxic properties of lipid content [7]. Due to their small particle size and lipoidal occlusive nature, these carriers amplify the concentration of lipophilic agents on the skin surface [8]. These carriers can encapsulate both lipophilic and hydrophilic drug molecules and provide controlled drug release and targeted drug delivery to specific cells, enhanced physical stability, good tolerability, and ease of scale-up [9]. The SLNs reduce water loss from the skin surface by forming a thin film that enhances the appearance of healthy human skin and reduces atopic eczema symptoms [10]. Chitosan, a natural polymer, is widely used in food, cosmetic, and drug delivery research due to its biocompatible and biodegradable nature and its antibacterial and wound-healing activities. When the SLNs are decorated with chitosan by physical adsorption, they can modify the physicochemical properties of the carriers by imparting a positive charge, which is derived from the protonated amino groups in chitosan, to their surface [11]. The use of chitosan in modification of nanocarriers can improve the stability of SLN dispersions and the degree of cellular interaction in biological models due to chitosan’s muco- and bioadhesive properties [12]. The SLN dispersions could be easily incorporated into their corresponding gel to achieve a semisolid consistency for skin application [13].

Solid lipid nanoparticles (SLNs) have proven efficacy in maximizing dermal drug concentration due to their lipoidal nature, capacity for higher drug encapsulation of class 11 drugs, biocompatibility, and safety when delivered topically [14]. These carriers are considered superior to other nanoparticulate carriers; they are physiological, able to hydrate the skin, and can be easily decorated with positively charged ligands like chitosan for improving the retention of drugs in the inflamed area of the skin [15,16]. Due to the potential advantages of SLNs in topical drug delivery, we formulated an immune suppressant drug, tacrolimus, as SLNs for potential application in atopic dermatitis.

Atopic dermatitis is a common inflammatory skin disorder with limited treatment options, affecting 10% of adults and 20% of children worldwide [17]. Tacrolimus is an immunosuppressant drug that belongs to the macrolide family, possessing prominent therapeutic efficacy in inflammatory conditions like atopic dermatitis [18]. Tacrolimus belongs to the BCS-II drug group due to its low solubility (4–12 μg/mL) and high permeability [19]. Tacrolimus cannot readily cross the stratum corneum due to its high molecular weight (822.95 g/mol) and strong lipophilicity (partition coefficient log P = 3.96 ± 0.83) [20]. The US-FDA approved ointment formulation (Protopic^®^; Astellas Pharma, Tokyo, Japan) for the treatment of atopic dermatitis has limitations, e.g., a sticky sensation due to its greasy nature. In previous studies, tacrolimus has been encapsulated in mesoporous silica nanoparticles functionalized with amino and phosphonate groups that improve its solubility sevenfold in comparison to free tacrolimus [21]. Significantly higher drug retention in the skin was achieved when the mesoporous silica nanoparticles were converted into Carbopol gel, suggesting that nanoparticle-loaded gel formulation could be a promising strategy for the topical delivery of tacrolimus. The goal of our study was to formulate tacrolimus-loaded SLNs that were coated with chitosan and loaded into gel formulations to prevent lipid degradation and drug leaching and provide controlled release of the drug for potential application in the treatment of atopic dermatitis.

## 2. Results and Discussion

### 2.1. Preparation of SLNs

The SLNs were fabricated using a solvent evaporation method from generally recognized as safe ingredients [22] as mentioned in Table 1. The components of the formulation include stearic acid, a low-cost, easily available solid lipid with high drug loading efficiency [23]. Polysorbate 80 and sorbitan monooleate were used as potent surfactants with desired HLB values for promoting colloidal stability [24]. Ethanol overall contributes to the homogeneity of SLNs. Chitosan, a natural coating polymer, was used as a decorating ligand for increasing drug penetration and promoting drug retention in inflamed tissue. Chitosan has controlled release properties with added drug loading and entrapment efficiency [25].

### 2.2. Characterization of Tacrolimus-Loaded SLNs Formulation

**Figure 1:**
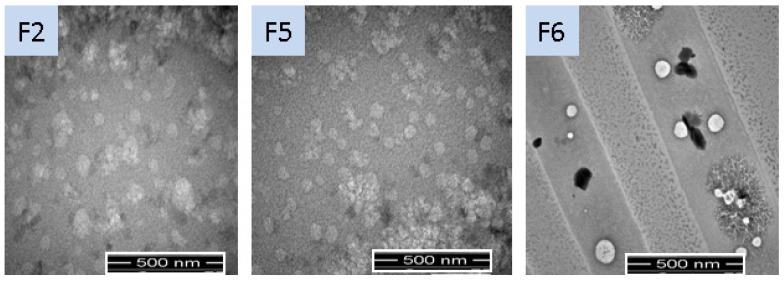
Morphology of optimized SLN formulations using TEM analysis.

**Figure 2:**
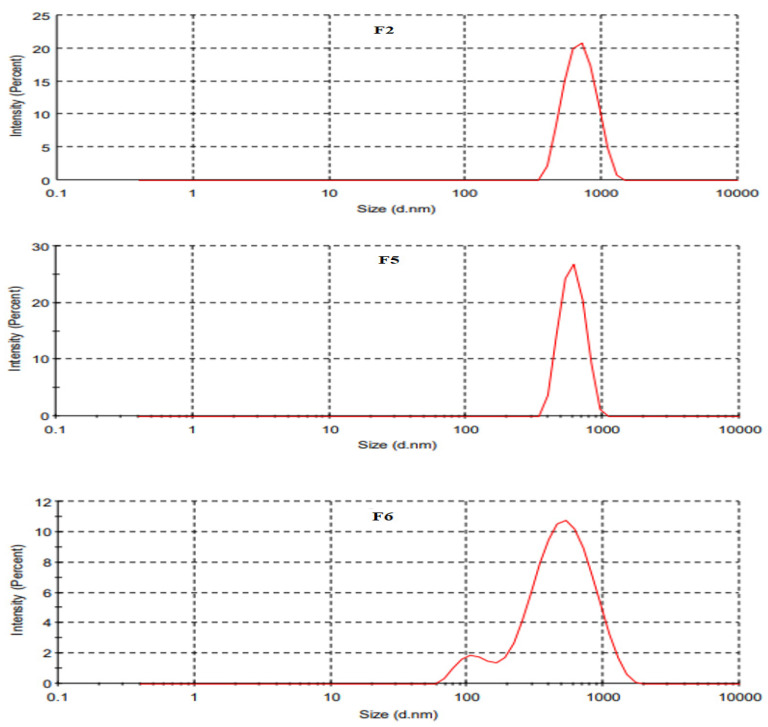
Particle size distribution of optimized SLN formulations using DLS analysis.

The physicochemical properties of SLNs were shown to have a significant impact on topical drug delivery [26] and are mentioned in Table 2. The particle size of uncoated SLNs was in the range of 439 to 669 nm, while coated SLNs had a droplet size of 523 ± 3.79 nm (Figure 1 and Figure 2). The particle size decreased from 669 ± 5.06 (F1) to 489 ± 6.81 (F2) with increasing concentration of Tween 80 due to lower interfacial tension, thereby improving miscibility between the layers of SLN dispersions. Any further increase in the concentration of Tween 80 in formulation resulted in an increasing trend of particle size from F2 to F3 due to the formation of micelles. At the critical micelle concentration, the surface becomes fully loaded with Tween 80 molecules, and thus beyond the critical micelle concentration, the interfacial tension change is nearly negligible resulting in the increased particle sizes [27]. Among Tween-80-based formulations, F2 was selected and further added with Span 80 for improved stabilization of dispersion. Addition of Span 80 in SLN formulations (F4 & F5) reduced the particle size significantly (Student *t*-test; *p* ≤ 0.05). The F5 was then coated with chitosan (F6), thereby increasing its particle size [28]. The PDI values of both coated and uncoated tacrolimus-loaded SLNs were in the acceptable range of <0.4, presenting the homogeneous dispersion system (Table 2). The PDI value of F6 was significantly lower than other formulations, which could be due to repulsive forces of the positive charges (-NH_2_) and additional surfactant action of chitosan [29]. The Zeta potential values ranged from −25.80 (F1) to −15.40 (F5) and reverted to a positive value in the case of chitosan-coated formulation (F6) due to the protonated amino group of chitosan, thereby confirming the successful decoration of SLN droplets [28]. The zeta potential values decreased from F1 to F5 due to increased nonionic surfactant concentration. The potential charges of all the tacrolimus-loaded SLNs showed good electrochemical stability due to electrostatic repulsion amongst particles [24]. The TEM analyses support the finding of photon correlation spectroscopy with respect to particle size and homogeneity. The particles were spherical and uniformly dispersed (Figure 1).

Lipid components and the nature of surfactants are the two main components of SLNs that can affect drug encapsulation efficiency [30]. At constant lipid concentration, both drug content and encapsulation efficiency increased with the increasing concentration of surfactant (Table 2). The lower drug entrapment efficiency values in the case of the first three formulations (F1, F2, and F3) were due to the fabrication of SLNs with only one surfactant (Tween 80). The longer alkyl chain of polysorbate 80 reduces the HLB value, which ultimately reduces the encapsulation efficiency [30]. The decreased entrapment efficiency could also be explained by the partition phenomenon, characterized by the increased partitioning of drug from inner phase to outer phase due to the presence of high surfactant concentration in the exterior phase; this supports an increased leakage of drug from internal to external phase [31,32]. The Span 80 with an HLB value of 4.3 has a higher affinity for lipids when compared to the Tween 80 with an HLB value of 15.0. The combination of two surfactants produces nanocarriers with higher stability [23]. The maximum entrapment efficiency of F6 could be attributed to reduced leakage of drug from stable SLN droplets (Table 2; [28]).

Healthy skin with a pH of approximately 4.9 to 5.9 owns acid/base assets, which might alter the mark of ionization of ionizable drug, ultimately affecting the drug permeation through the skin. The pH of the topical formulation is a significant factor regarding solubility and consistency of the drug product throughout the storing period [33]. The pH of prepared SLNs was in the range of 5.45 to 5.53 as tabulated in Table 3, depicting their suitability as topical formulation [34]. The viscosity of tacrolimus-loaded SLNs ranged from 11.56 to 39.87 (Table 3). It has been previously elaborated that the viscosity of SLNs is the function of lipid component, type and quantity of surfactant, and the quantity of aqueous phase [21]. The viscosity increased with the increasing concentration of surfactant. The addition of chitosan has also been shown to have a prominent effect on the viscosity of the formulation, thereby increasing the contact of nanoparticles with the skin surface and ultimately affecting the skin retention and permeation properties of the topical formulation [24,25]. The relations between two immiscible ingredients are governed by surface tension [35]. The surface tension of the tacrolimus-loaded SLNs decreased with increasing surfactant concentration (Table 3). The increased surfactant concentration minimizes the superficial tension and thereby reduces the surface area of each particle in the formulation. The minimum surface tension observed for F6 was due to the presence of double surfactant with additional surfactant properties of chitosan that could probably increase the retention and permeation of the tacrolimus. The specific gravity and density of all tacrolimus-loaded SLNs were close to that of deionized water.

### 2.3. Drug Release

**Figure 3:**
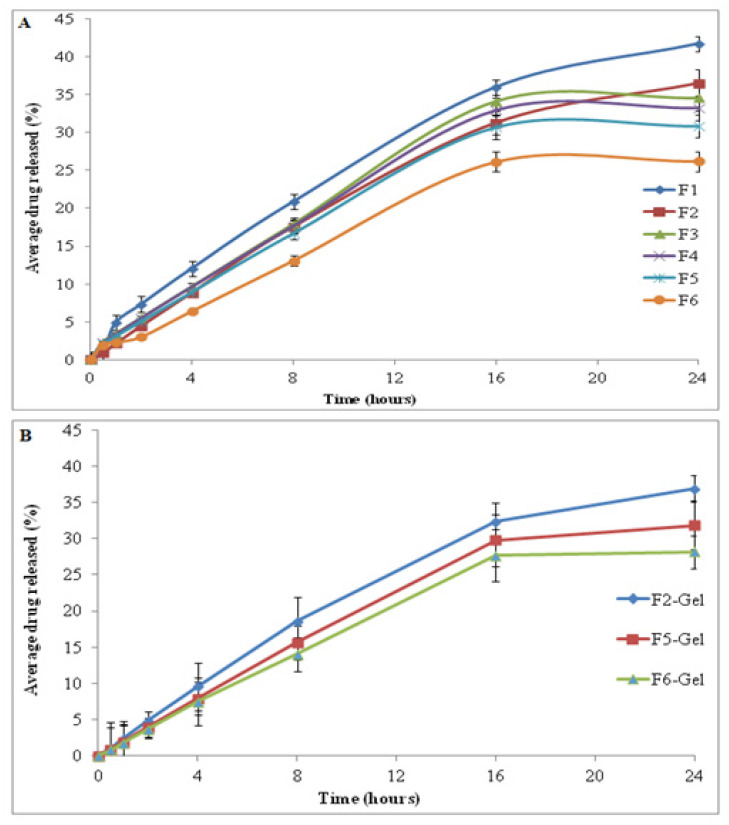
Drug release pattern from (**A**) SLN formulations and (**B**) SLN-loaded gel.

The in vitro drug release, being a valuable indicator of in vivo drug performance, was performed at skin temperature of 32 ± 2 °C using a modified Franz diffusion cell. The cellulose acetate membrane with a pore size of 0.45 µm was clamped between the donor and recipient compartment of the Franz diffusion cell. The release of drug from the SLNs was biphasic; a burst drug release was followed by controlled release of drug. The initial abrupt release of drug could be due to the availability of drug at the interphase and in the hydrophilic phase of SLNs. The release of drug was comparatively retarded from F1 to F5 due to increased viscosity and higher surfactant concentrations (Figure 3A; Table 3). The release of drug from the chitosan-coated formulation was significantly retarded when compared to uncoated SLNs due to decreased solubility of chitosan at physiological pH and the higher viscosity (ANOVA, *p* ≤ 0.05; [36]. Another reason could be the enhanced drug entrapment efficiency of chitosan-coated SLNs [37]. When the SLNs were converted to their corresponding gel formulations using sodium alginate as a gelling agent, the release of drug was significantly retarded due to presence of polymeric matrix as mentioned previously (Figure 3B; [38]).

### 2.4. Drug Permeation through the Rats’ Skin

**Figure 4:**
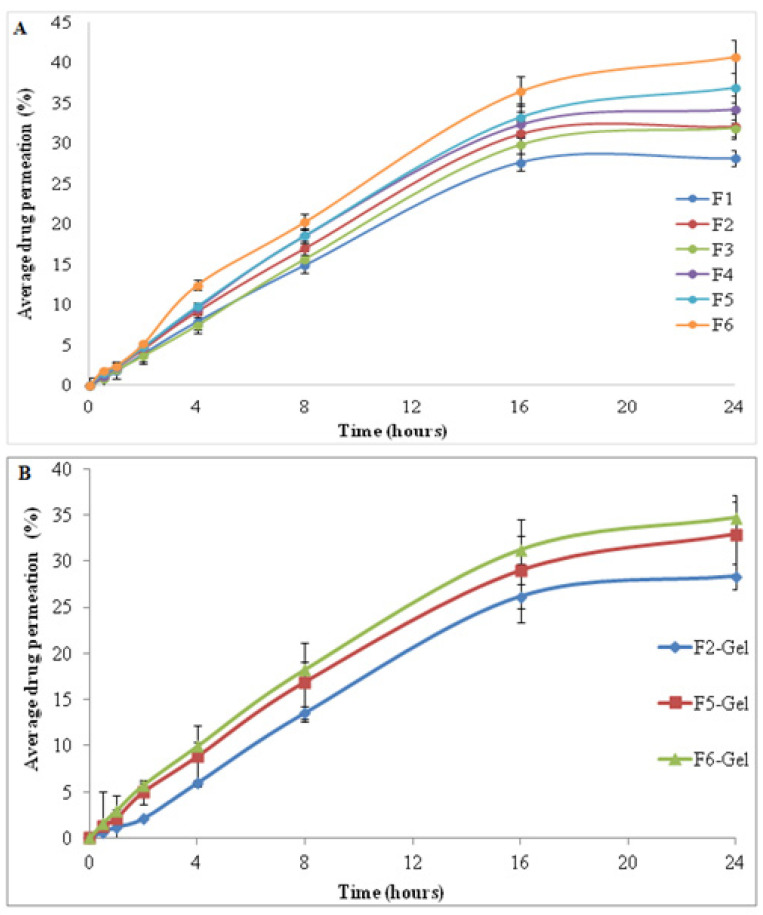
Permeation of drug through the skin from (**A**) SLNs formulation and (**B**) SLN-loaded gel.

The permeation of tacrolimus through the full thickness skin of Sprague dawley rats from applied SLNs and their corresponding gel formulation is shown in Figure 4A,B, respectively. The total amount of drug permeated linearly increased from F1 to F6 due to decreased surface tension. The increasing concentration of surfactant and decreased Zeta potential values could also result in improved drug permeation from F1 to F5. The innate features of SLNs, including compatibility with the dermal lipids, the occlusive effect, and their lesser particulate size may also be held responsible to improve the capacity of SLNs to permeate the skin [39,40]. The minor change among the ordinary particle dimensions of the prepared formulations was not the main aspect that inclined the drug permeation into the skin; it was the thermodynamic properties that caused the discharge at a topical temperature [41]. The higher drug permeation in the case of chitosan-coated SLNs (F6) could be due to the interaction of positively charged chitosan and negatively charged stratum corneum of the skin [37]. In response to this interaction, secondary changes occurred in the skin with the resulting disorganization of lipids and opening of skin pores that facilitate the drug penetration by intercellular and/or transcellular pathways [42]. Tween 80, a hydrophilic surfactant, hydrates the stratum corneum via lipid fluidization with water [43]. The permeation of drug from SLN-loaded gel is shown in Figure 4B It can be observed that the permeated percentage was lower than the nongel formulation. This retarding infiltration of the drug from the gel formulation could be due to the higher viscosity thereby decreasing the skin drug diffusion within tested time duration. However, the gel formulation increases the overall retention of drug within the skin layers [37,44].

### 2.5. Skin Drug Retention

**Figure 5:**
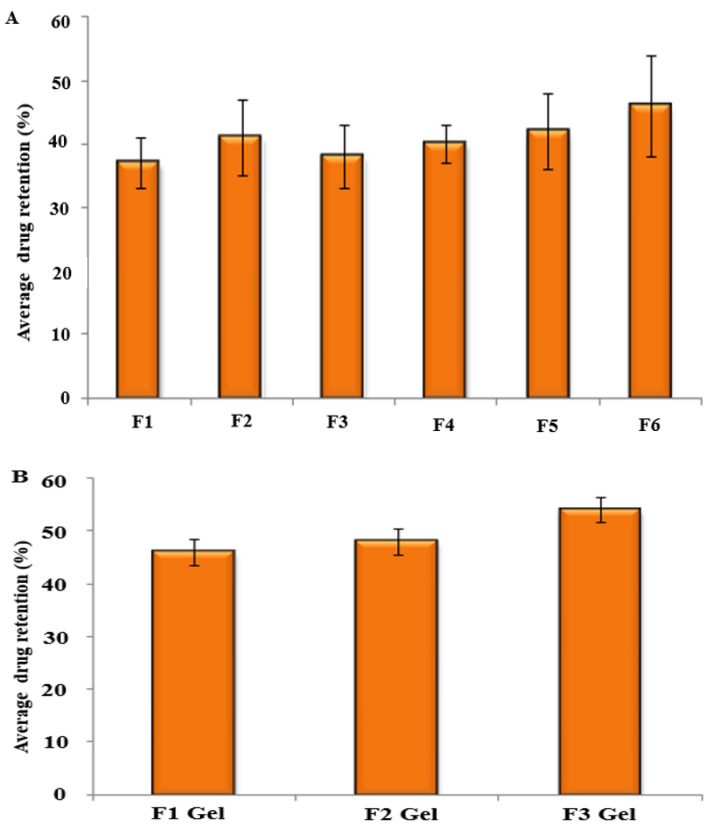
Drug retention profile in the skin from (**A**) SLN formulations and (**B**) SLN-loaded gel.

The amount of drug retained in the skin was estimated by extracting the drug from the skin tissue. The retention of drug within intact skin from SLNs was comparatively higher for the chitosan-coated formulation than noncoated carriers due to its bioadhesive properties. However, the change was not significant (Figure 5A; F2 to F6; *p* ≥ 0.05). The higher retention of drug in the case of chitosan-coated SLNs was due to the formation of a dense occlusive layer of solid lipid that melts and penetrates through the skin due to suitable physicochemical properties of SLNs and the penetration enhancement effect of chitosan [45]. When skin drug retention of SLNs was compared to their corresponding gel, the latter achieved significantly higher drug retention (*p* ≤ 0.05; Figure 5B). This higher retention of gel formulations could be attributed to the penetration enhancement effect of the gelling agent that reduces water loss from the skin, thereby producing hydration for improved retention of drug within the skin [44,46].

## 3. Conclusions

Tacrolimus, a drug of choice in atopic dermatitis, was successfully formulated in SLNs, having suitable physicochemical properties for skin drug application. The SLNs achieved optimum permeation ranging from 25 to 40%. The release of drug from all formulations was controlled; however, the addition of chitosan sustained drug release significantly. The total amount of drug permeated increased linearly from F1 to F6 due to the increasing concentration of surfactants thereby reducing surface tension and promoting skin drug interaction. Conversion of SLNs into their corresponding gel improved skin drug retention, thereby achieving the goal of topical drug application in the management of atopic dermatitis.

## 4. Materials and Methods

### 4.1. Materials

The tacrolimus was kindly gifted to us by Siam Pharmaceuticals (Pvt) Ltd., Islamabad, Pakistan. Stearic acid (Sigma Aldrich, St. Louis, MO, USA) was used as a solid lipid while polysorbate 80 and Sorbiton monooleate 80 (Sigma Aldrich, St. Louis, MO, USA) were used as surfactants. Ethanol (Merk, Kenilworth, NJ, USA) was used as solvent, and chitosan (LMW; Sigma Aldrich, St. Louis, MO, USA) was utilized as a penetration enhancer. Sodium alginate and glycerol along with humectant trietanolamine were used as gelling agents. All other chemicals used in the study were of pharmaceutical grades and were used without any purification.

### 4.2. Preparation of Tacrolimus-Loaded SLNs

The tacrolimus-loaded SLNs were prepared using varying concentrations of surfactants and cosurfactants via a solvent emulsification technique as mentioned previously [25]. Briefly, the stearic acid was melted on a water bath (Memmert WPE 45, Schwabach, Germany) at 70 ± 2 °C for 90 min. The tacrolimus previously dissolved in ethanol was mixed with melted lipid with constant magnetic stirring for 15 min at 70 ± 2 °C. The aqueous phase of SLNs was prepared by adding Tween 80 to distilled water with continuous magnetic stirring at 70 ± 2 °C for 60 min. To improve the stability of colloidal dispersion, Span 80 was also added in some formulations (F4–F6). The final dispersion was then achieved by mixing the two phases at constant magnetic stirring for 2.0 h at 70 ± 2 °C. The final formulation was subjected homogenization at 10,000 rpm (Ultra-turrax, DXT45) for 5 min followed by solvent evaporation. The optimized formulation (F5) was coated with 85% deacetylated chitosan and had a molecular weight of 50,000 to get the final formulation (F6) as mentioned in Table 1A.

### 4.3. SLN-Loaded Gel Preparation

The optimized SLNs formulations (F2, F5 and F6) were converted into a gel by the addition of glycerol and sodium alginate to the dispersion. Trietanolamine was also added as a humectant upon continuous stirring, which yielded the desired gel. The prepared gel incorporated with the drug-loaded SLNs was preserved in the refrigerator at 2–8 °C until used. The composition of prepared SLNs and their respective gel formulations is shown in Table 1A,B respectively.

### 4.4. Physicochemical Characterization

The SLN colloidal dispersions were evaluated for their size and size distribution (PDI), surface charge, morphology, drug content, surface tension, viscosity, pH, and density to predict their suitability for topical application.

#### 4.4.1. Particle Size and Polydispersity Index

The particle size and particle size distribution were investigated at 25 ± 1 °C using a Zetasizer (Malvern Instruments, Worcestershire, UK). In brief, 10 µL of the sample was added to 1 mL of deionized water, mixed well, and subjected to vortex mixing for 2 min followed by photon correlation spectroscopic analysis as mentioned previously [47]. The results obtained were recorded in triplicate and the average particle size and PDI values were tabulated.

#### 4.4.2. Zeta Potential

The surface charge on prepared SLN colloidal dispersions was evaluated using Zetasizer (Malvern Instruments, Worcestershire, UK). An aliquot of 700 µL nondiluted colloidal dispersion was added to Zeta potential cell and evaluated for its charge in triplicate as mentioned previously [48].

#### 4.4.3. Structural Analysis

Transmission electron microscopy (TEM) at specified magnification was used to evaluate the facet shape and structural features of the particulates. A double-sided adhesive tape adhered to the aluminium stub was used for the placement of dispersion drops followed by vacuum drying. The drops were then coated with the thin layer of gold and the images were taken at suitable magnification as previously mentioned [49].

#### 4.4.4. Drug Encapsulation Efficiency

The drug content and encapsulation efficiency of the SLN dispersions was investigated spectrophotometrically. Briefly, 1.5 mL of the sample was added to centrifuge tubes and centrifugated at 14,000 rpm for 10 min using a centrifuge machine (D3024, SCILOGEX, Rocky Hill, CT, USA). The supernatant layer was analyzed with a UV spectrometer (UV-160, Shimadzu, Kyoto, Japan) for free drug content after appropriate dilution with phosphate buffer pH 7.4 at λ max 294 nm. The entrapped drug in the SLN droplets was determined by first dissolving the sediment layer in ethanol followed by dilution and vortex mixing for 10 min [50]. The encapsulation efficiency of prepared SLNs was then calculated as mentioned in Equation (1):

Encapsulation efficiency = (Total drug content-free drug content)/(Total drug content) × 100(1)

#### 4.4.5. Density

The density of prepared SLNs was determined using an ordinary pycnometer at 25 ± 1 °C. Initially the weight of the empty pycnometer of known volume (V) was recorded as W1, followed by the weight of the water-filled pycnometer (W2). The weight of the SLN-filled pycnometer was labeled as W3. The density of SLNs was calculated using the following equation [48].

Density of water = W2/V(2)

Density of SLNs = W3/V(3)

Specific gravity of SLNs = (SLNs density)/(Water density)(4)

#### 4.4.6. Surface Tension

A laboratory stalagmometer was used for measuring the surface tension of the SLN dispersions. The instrument was cleaned with un-ionized water and filled to the point A with double distilled water. The filled water was then run down drop wise to the point B while recording the number of drops. Similarly, same steps were repeated for the SLN dispersions. The density of SLNs was already calculated as mentioned above in Section 4.4.5. The surface tension of SLNs was then calculated using Equation (5) as mentioned previously [51].

^γ^1 = (^δ^1 ∗ n1 ÷ ^δ^2 ∗ n2) ∗ ^γ^2 (5)

where:

^γ^1: Surface tension of dispersion; 

^γ^2: Surface tension of water;

^δ^1: Density of dispersion;

^δ^2: Density of water;

n1: Number of drops of SLNs; 

n2: Number of drops of de-ionized water.

#### 4.4.7. Viscosity

The viscosity of SLN dispersions was determined using an Ostwald viscometer at 25 ± 1 °C (Poulten selfe & Lee Ltd., Essex, UK). The instrument was washed, dried, and fixed vertically on the stand. The apparatus was filled with distilled water up to mark A. The distilled water was allowed to flow down to mark B, and the time of flow between the two points was recorded. Similarly, water was replaced with SLN dispersions, and the time of flow from point A to B was noted. The final viscosity of SLNs was calculated using equation 6 [51]. The readings were recorded for three consecutive experiments and the values were shown as mean ± SD.

ƞ1 = (d1 ∗ t1 _ d2 ∗ t2) ∗ ƞ2(6)

where:

ƞ1: Viscosity of the SLNs sample

ƞ2: Viscosity of the pure distilled water

t1: Flow time of prepared SLNs sample

t2: Flow time of pure distilled water

d1: Density of SLNs sample

d2: Density of pure distilled water

#### 4.4.8. pH

A commonly used pH meter (Accumet meter and Denver instruments) was utilized at 25 ± 1 °C for predicting the pH of the SLNs. A known buffer solution with a pH of 7 was used to calibrate the instrument. After calibration, the pH of the SLN dispersions was determined and tabulated as mean ± SD of triplicate readings.

### 4.5. Ex Vivo Drug Release

The release of drug from designed SLNs and their corresponding gel was determined using Franz diffusion cell (PRIME GEAR Inc. NO 4G 1-22-3-12, Delhi, India). The cellulose acetate membrane (0.45 µm) was fixed between the donor and receptor compartment. The receptor compartment was filled to its capacity with acetate buffer pH 5.5 at 32 ± 2 °C to mimic the skin conditions. The magnetic stirring was stabilized at 600 rpm to keep sink condition. The donor compartment was fed with 1 gm of the sample. An aliquot of 0.5 mL was withdrawn from the receptor compartment through the sampling port with the help of a calibrated syringe at the time intervals of 0, 0.5, 1, 2, 4, 8, 16 and 24 h. The samples were analyzed using a UV-visible spectrophotometer (UV-1601 Shimadzu, Kyoto, Japan) at λ max of 294 nm [52]. The cumulative release of tacrolimus from the designed carrier system was calculated using the standard calibration curve. All the results were recorded as the mean of triplicate experiments. 

### 4.6. Animal Ethics Approval

The approval of animal use in research and procedures to be carried out for isolation of skin from Sprague dowley rats was granted from Quality Enhancement Cell (QEC), Gomal University, D.I. Khan Khyber Pakhtunkhwa, Pakistan.

### 4.7. Ex Vivo Drug Permeation and Retention

The permeation of drug through the dead keratinized layer of the skin (stratum corneum) and its potential deposition in desired tissue of the body is a critical factor for the success of topical and transdermal drug delivery. The drug permeation of the designed carriers through the rats’ (Sprague dawley) skin was evaluated using Franz diffusion cells (PermeGear, Inc., Hellertown, PA, USA). The Franz diffusion cell consists of 6 cells with a diffusional surface area of 0.654 cm^2^ and a receptor cell volume of 5 mL, operated at continuous stirring mode (600 rpm) at 37 °C. The rat weighing 200 ± 20 g was sacrificed by the cervical dislocation method, followed by complete hair removal from the abdominal area using a razor. The full-thickness skin was surgically excised from Sprague dawley rats as per approved protocols in compliance with the Ethical Review Committee, Gomal University, D.I. Khan, Pakistan. The excess fats were removed, washed with normal saline and stored at −20 °C for future experimental use. The skin was clamped between the donor and recipient compartment of the Franz diffusion cell. To maintain sink conditions, phosphate buffer 7.4 was added to the volume of the receptor compartment. The temperature and magnetic stirring were kept constant at 37 ± 2 °C and 600 rpm, respectively. The SLN’s, having a known quantity of the drug, were fed to the contributor chambers of the apparatus. An aliquot of 0.5 mL was withdrawn from the receptor compartment through the sampling port with the help of a calibrated syringe at the time intervals of, 0, 0.5, 1, 2, 4, 8, 16, and finally 24 h. The deficiency of withdrawal sample was top-up using the same quantity of fresh buffer. The drug content in each sample was determined using a UV-visible spectrophotometer at λ max of 294 nm. 

Following the permeation experiment, the percentage of drug retained in the skin was determined by isolating the mounted skin on the Franz diffusion cell, carefully weighed and cut into pieces. The cut pieces of the isolated skin were extracted with 10 mL of methanol by mechanically shaking in a water bath (BW-20G, Lab. Companion, Billerica MA, USA) at 37 ± 1 °C overnight. To further improve the extraction efficiencies, tissue cell lysis reagent was introduced at a ratio of 1:20 and sonicated (EUROSTAR digital, IKA^®^, Kuala Lumpur, Malaysia) for 30 min. All the samples were harvested by ultracentrifugation (12,000 rpm) at 10 °C for 20 min and quantification of tacrolimus in the rats’ skin was performed using a UV-visible spectrophotometer as mentioned previously [53].

### 4.8. Statistical Analysis

All the values were tabulated as a mean ± SD for triplicate experiments. The data was statistically evaluated using one way ANOVA or paired t-test and a *p* value of < 0.05 was considered statistically significant.

## Figures and Tables

**Table 1:** (**A**): Composition of drug-loaded SLNs formulations; (**B**): Composition of optimized SLN gel formulation.

**Sample**	**Tacrolimus** **(gm)**	**Tween 80** **(gm)**	**Span 80** **(gm**)	**Stearic Acid** **(gm)**	**Ethanol** **(gm)**	**Chitosan** **(gm)**	**Distill Water** **(gm)**
F1	0.1	1	----	1	10	----	87.90
F2	0.1	1.25	----	1	10	----	87.65
F3	0.1	1.50	----	1	10	----	87.40
F4	0.1	1.25	0.25	1	10	----	87.40
F5	0.1	1.25	0.50	1	10	----	87.15
F6	0.1	1.25	0.50	1	10	0.0015	87.14
**Sample**		**Sodium Alginate (g)**		**Glycerol (g)**		**Trietanolamine (g)**	
Gel (F2)		1		5		1	
Gel (F5)		1		5		1	
Gel (F6)		1		5		1	

**Table 2:** Physicochemical characteristics of the SLNs.

Formulation Code	Size (nm)	PDI	Zeta Potential (mV)	Drug Content (mg/mL)	Entrapment Efficiency (%)
F1	669 ± 5.06	0.302	−25.80 ± 0.05	0.86 ± 0.03	68.95 ± 0.03
F2	489 ± 6.81	0.318	−23.10 ± 0.02	0.89 ± 0.02	72.26 ± 0.05
F3	639 ± 8.43	0.342	−20.20 ± 0.04	0.87 ± 0.04	69.71 ± 0.02
F4	578 ± 4.12	0.358	−19.30 ± 0.02	0.88 ± 0.03	78.38 ± 0.04
F5	439 ± 4.44	0.372	−15.70 ± 0.02	0.89 ± 0.02	80.45 ± 0.05
F6	523 ± 3.79	0.292	17.40 ± 0.07	0.91 ± 0.03	83.68 ± 0.04

**Table 3:** Viscosity, specific gravity, Surface tension, pH, and density of prepared SLN formulations.

Formulation Code	Viscosity	Surface Tension (Dynes/cm^2^)	pH	Specific Gravity	Density
F1	11.56 ± 0.43	30.82 ± 0.81	5.45 ± 0.14	0.977 ± 0.09	0.947 ± 0.02
F2	14.22 ± 0.22	28.33 ± 0.78	5.36 ± 0.05	0.984 ± 0.04	0.952 ± 0.04
F3	23.11 ± 0.26	27.66 ± 0.49	5.44 ± 0.07	0.991 ± 0.06	0.961 ± 0.04
F4	25.87 ± 0.55	22.79 ± 0.63	5.11 ± 0.12	0.992 ± 0.01	0.979 ± 0.03
F5	36.95 ± 0.54	21.43 ± 0.52	5.29 ± 0.14	0.993 ± 0.02	0.988 ± 0.03
F6	39.87 ± 0.43	19.81 ± 0.22	5.53 ± 0.07	0.996 ± 0.03	0.997 ± 0.05

## Data Availability

Requests to access the datasets should be directed to the corresponding author.
